# Tannin Phytocomplex Improves IBS‐D Symptoms and Gut‐Brain Markers: Prospective Pilot Study

**DOI:** 10.1002/mnfr.70451

**Published:** 2026-04-20

**Authors:** Silvia Molino, María M. Piskorz, Alberto Lerma‐Aguilera, Fabiana López Mingorance, Cielo Gutierrez, Juan M. Montero, Tatiana Uehara, Harumi Hashimoto, Esteban González Ballerga, Jorge A. Olmos

**Affiliations:** ^1^ Silvateam Spa R&D Unit San Michele Mondovì Italy; ^2^ Universidad de Buenos Aires Hospital de Clínicas José de San Martin Sector Neurogastroenterología del Servicio de Gastroenterología Buenos Aires Argentina; ^3^ University of Lund Neuroinflammation Lund Sweden; ^4^ Universidad de Buenos aires/ IBIMOL Hospital de Clínicas José de San Martin Programa de Estudios Pancreáticos Buenos Aires Argentina; ^5^ Universidad de Buenos Aires Hospital de Clínicas José de San Martin Servicio de Gastroenterología Buenos Aires Argentina

**Keywords:** Clinical Pilot study, Gut‐Brain Axis, Irritable Bowel Syndrome, Short Chain Fatty Acids, Tannins

## Abstract

Diarrhea‐predominant irritable bowel syndrome (IBS‐D) is a multifactorial disorder involving gut‐brain axis dysregulation, altered barrier integrity, microbial imbalance, and stress‐related mechanisms, with limited nutritional strategies targeting these pathways. Tannin‐rich extracts may exert barrier‐protective, microbiota‐modulating, and stress‐related effects.

In a prospective before‐after pilot study (NCT05207618), 28 adults with IBS‑D completed a 60‑day tannin‐based supplementation (480 mg/day). Outcomes included IBS Severity Scoring System (IBS‐SSS), bowel habits, somatization, perceived stress, serum zonulin, faecal short‐chain fatty acids, and hydrogen breath test. Linear mixed models assessed changes over time.

IBS‐SSS decreased by an estimated mean of 70 points (95% CI: 34–107; *p* < 0.0001); stool consistency improved by 1.38 (95% CI: 0.72 to 2.05; *p* < 0.0001), and bowel movements/day decreased by 0.84 (95% CI: 0.08 to 1.71; *P* = 0.028). Somatization and stress scores declined significantly (both *P* ≤ 0.003). Zonulin dropped (*P* < 0.0001), butyrate increased (*p* = 0.001), and propionate decreased (*p* = 0.017). Hydrogen‐positive subjects at baseline (3/28) were all negative at 60 days. No adverse events occurred.

This pilot suggests a tannin‐based phytocomplex may improve gastrointestinal and psychological symptoms in IBS‐D, with biomarker changes indicating enhanced barrier function and microbial metabolism. Larger trials are warranted.

## Graphical abstract

In adults with IBS‐D, a 60‐day tannin‐based supplementation was associated with improvements in gastrointestinal scores, stool patterns, and psychological measures. Mechanistic readouts showed lower zonulin, SCFA shifts toward butyrate, and normalization of hydrogen breath tests, supporting a broad, barrier, and microbiota‐oriented intervention profile.

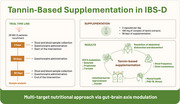

**Abbreviations**
BM/Day: Bowel movements/day
BSFS: Bristol stool form scale
HADS: Hospital anxiety and depression scale
IBS: Irritable bowel syndrome
IBS‐D: Diarrhea‐predominant irritable bowel syndrome
IBS‐SSS: Irritable bowel syndrome severity scoring system
PHQ‐15: Patient health questionnaire‐15
PSS: Perceived stress scale
SCFA: Short‐chain fatty acid

## 1 Introduction

Irritable bowel syndrome (IBS) is a chronic functional gastrointestinal disorder involving the gut–brain axis and affects 5%–10% of the population [1]. Among the Rome IV subtypes [2], diarrhoea‐predominant IBS (IBS‐D) is the most prevalent and is characterized by recurrent abdominal pain associated with changes in stool frequency and form.

The gut–brain axis, integral to the pathophysiology of IBS constitutes a sophisticated bidirectional communication network linking the gastrointestinal tract with the central nervous system via neural, hormonal, and immune mechanisms. This ongoing interaction, in which each component influences the other, underscores the importance of a comprehensive management strategy that addresses both the physiological and psychological dimensions of IBS [3, 4].

Building on this concept, the disruption of gut–brain signalling in IBS is driven by multiple interacting factors, making its pathophysiology highly complex. IBS pathophysiology is multifactorial, involving dysbiosis, small intestinal bacterial overgrowth (SIBO), dysmotility, visceral hypersensitivity, and psychosocial distress [4, 5]. These alterations are associated with molecular changes, including alteration of epithelial tight junctions and activation of inflammatory pathways (e.g., NF‐κB), leading to increased intestinal permeability and low‐grade mucosal inflammation. Current treatments, such as dietary interventions, antidiarrheal drugs, and psychological therapies, primarily target symptom relief rather than underlying mechanisms [6, 7, 8].

Tannins, polyphenolic secondary metabolites produced by plants, have traditionally been studied for their antidiarrheal properties [9]; however, emerging evidence suggests a broader mechanistic potential relevant to gut–brain axis modulation. Specifically, tannins can attenuate inflammatory signalling, enhance epithelial barrier integrity by upregulating tight junction proteins (e.g., occludin, claudins), and influence microbiota composition and short‐chain fatty acid (SCFA) profiles [10, 11, 12, 13]. These actions may indirectly affect neuroimmune pathways and stress‐related symptom expression, positioning tannins as promising candidates for multi‐target intervention in IBS‐D. Recent studies highlight also the ability of tannins, to restore mucosal barrier function and dampen low‐grade inflammation, supporting the chance to investigate these phytocomplexes as a novel nutritional strategy for disorders of gut–brain interaction [14, 15].

To our knowledge, no previous clinical trial has evaluated a tannin‐based phytocomplex in IBS‐D patients with simultaneous assessment of gastrointestinal symptoms, psychological outcomes, and mechanistic biomarkers such as zonulin and SCFAs.

This prospective, single‐arm, open‐label before‐after study was designed to preliminary evaluate tolerability and explore efficacy of a standardised quebracho (*Schinopsis lorentzii*) and chestnut (*Castanea sativa*) tannin‐rich extract on gastrointestinal symptoms in IBS‐D patients. Additionally, secondary outcomes encompassed investigation of patient's mental health outcomes, mechanistic effects on gut permeability, inflammation, and microbiota‐related metabolites, as well as evaluation of correlations with clinical results.

## 2 Experimental Section

### 2.1 Food Supplement

The functional ingredient was a standardized blend of tannin‐rich phytocomplexes from quebracho (*Schinopsis lorentzii Engl*.) wood and chestnut (*Castanea sativa Mill*.) extracts (Silvateam S.p.A.), obtained by food‐grade hot water extraction. The dietary supplement contained 2.98 mmol gallic acid per gram (GEAC Folin–Ciocalteu method, [16]), with at least 65% tannins confirmed by OIV testing [17]. Quebracho extract is characterised by the presence of condensed tannins (profisetinidins, fisetin) [18], while chestnut extract contains mainly ellagitannins (digalloyl‐glucose, glucose, gallic acid) [19]. Capsules contained 240 mg of the blend plus 0.72 µg vitamin B12, administered twice daily (total 480 mg/day).

### 2.2 Study Design

This exploratory, single‐group, open‐label, before–after prospective study was conducted at University Hospital of Universidad de Buenos Aires “Hospital de Clínicas Jose de San Martín” (Argentina). The protocol was approved by the Ethics Committee (0979/19), registered at ClinicalTrials.gov (NCT05207618) [20], and complied with the Declaration of Helsinki.

Participants were thoroughly briefed about the study both verbally and in writing before providing their written consent. After screening, individuals in the trial ingested two capsules daily for 60 days. Adherence and adverse events were monitored by daily phone calls.

Assessments were performed at baseline (T0), 30 days (30d), and 60 days (60d) using validated questionnaires: IBS‐Severity Scoring System (IBS‐SSS), Hospital Anxiety and Depression Scale (HADS), Perceived Stress Scale (PSS), Patient Health Questionnaire‐15 (PHQ‐15), Bristol Stool Form Scale (BSFS), Bowel Movements/Day (BM/Day) and IBS symptom Likert scales. Blood and stool samples were collected at T0 and 60d. Venous blood was drawn into Vacutainer clot‐activator tubes, centrifuged (3000 g, 15 min, 4°C) to separate serum, while stools were placed in polypropylene containers. Both were stored at −80°C until further analysis. Hydrogen breath test was performed at both time points. No specific diet was prescribed; use of supplements or unlisted drugs was prohibited.

### 2.3 Study Patients

31 adults (18–80 years) with IBS‐D per Rome IV criteria were enrolled [21]. Inclusion required the presence of IBS symptoms for a minimum duration of three months, with symptom onset at least six months prior to evaluation. Participants also needed to report recurrent abdominal pain occurring at least once per week, associated with changes in defecation, bowel movement frequency, or stool form.

Exclusion criteria included ages < 17 and > 80 and subjects with intestinal symptoms who did not meet the specified criteria were excluded. All participants were not pregnant or lactating and did not use substances of abuse (including caffeine, tobacco, alcohol, or drugs) or medications affecting intestinal function. Anyone who had taken antibiotics within the previous four weeks or in the past six months (depending on treatment intensity and duration) was also excluded.

Patients were considered ineligible if they met any of the following: prior gastrointestinal surgery, severe comorbidities (neurological disorders, HIV/AIDS), diabetes mellitus, cirrhosis, celiac disease, major systemic illnesses (severe heart, kidney, or liver failure), inability to care for themselves, cognitive impairment affecting questionnaire responses, unwillingness or inability to attend the facility, or allergy to study product ingredients. Subjects with other pathologies were included only if deemed compatible by the investigating doctor.

All participants underwent routine labs, celiac serology, upper endoscopy with duodenal biopsies, and colonoscopy to exclude other causes of diarrhea.

### 2.4 Primary Outcomes

Three co‐primary outcomes were chosen to capture the global and bowel‑habit dimensions of IBS‑D: IBS‐Severity Scoring System (IBS‐SSS), Bristol Stool Form Scale (BSFS), and Bowel Movements/day (BM/day).

IBS‐SSS is validated questionnaire assessing abdominal pain, bowel dysfunction, and general well‐being, categorizing symptom severity as mild (75–<175), moderate (175–<300), or severe (≥ 300). For clinical interpretability minimal clinically important difference (MCID) of ≥ 50 points was also reported [22]. Stool consistency was evaluated using the BSFS, which rates stools from hard/lumpy to soft/watery on a seven‐point scale. Bowel movement frequency was tracked daily.

### 2.5 Secondary Outcomes

Secondary outcomes included global and specific symptom severity, psychological symptoms, serum zonulin, faecal short‐chain fatty acids, cytokine profile, and hydrogen breath test.

#### 2.5.1 Intestinal Symptoms

The global overall symptom severity was rated on a 7‐point Likert scale from 1 (no discomfort) to 7 (severe discomfort requiring rest and limiting daily activities). The same scale measured abdominal pain and distension severity.

*Psychological symptoms*: Improvements were assessed using self‐report questionnaires: the PHQ‐15 for somatic symptoms (score: 0–30; ≥ 5 mild, ≥ 10 moderate, ≥ 15 severe), HADS for anxiety and depression (two subscales, score: 0–7 normal, 8–10 borderline, 11–21 abnormal), and PSS for perceived stress (score: 0–13 low, 14–26 moderate, 27–40 high). These tools evaluate physical symptoms, emotional states, and stress levels in response to treatment.

#### 2.5.2 Serum Cytokines Analysis

Serum samples were analysed for 27 human cytokines, chemokines, and growth factors using the Bio‐Plex Pro Human Cytokine 27‐plex Assay (Bio‐Rad Laboratories, USA) according to kit instructions. Briefly, serum samples and standards were incubated with magnetic beads coated with capture antibodies, followed by biotinylated detection antibodies and streptavidin‐phycoerythrin. After washing, the beads were analyzed on a Luminex‐based reader, and concentrations were calculated from standard curves.

#### 2.5.3 Intestinal Permeability

The analysis was conducted by measuring zonulin family peptides in stool samples in vitro using the IDK Zonulin ELISA kit (Immundiagnostik AG, Germany), following the manufacturer's protocol. The assay showed intra‐assay variation ranging from 3.3% to 6.4%, and inter‐assay variation between 13.1% and 18.3%. In brief, 100 µL of standards, controls, and samples were placed into pre‐coated microtiter wells, followed by incubation first with biotinylated tracer and then with peroxidase‐conjugated streptavidin. After washing, substrate was added, and absorbance was read at 450 nm.

#### 2.5.4 SCFA Analysis

200 mg of faecal sample was diluted in sterile distilled water (1:4 to 1:8 w/v), vortexed for 1 min, and centrifuged at 10,000 g for 10 min. The supernatant was filtered (0.22 µm nylon membrane) and analysed by HPLC (Ultimate 3000 RSLC, Thermo Scientific) using a sulfuric acid mobile phase (pH ∼2, 0.6 mL/min) on an Aminex HPX‐87H column (300 mm × 7.8 mm, 9 µm) with UV detection at 210 nm for 23 min. Acetic, propionic, butyric, and formic acids were quantified against standards (1–2500 ppm) and reported as mM.

#### 2.5.5 Hydrogen Breath Test

After fasting for 8 h, participants consumed 75 g of glucose in 200 mL water. Breath samples were collected at baseline and every 15 min for 2 h, using a Gastrolyzer (Bedfont Scientific Ltd, England). Bacterial overgrowth was indicated by a hydrogen concentration increase over 20 ppm above baseline within 90 min, as previously described procedures [23].

### 2.6 Tolerability

Participants were monitored for adverse events throughout the study. Those with known allergies to supplement ingredients were excluded.

### 2.7 Statistical Analysis

Statistical analyses were conducted in R (v4.2.2) with figures generated via ggplot2. Continuous variables are presented as mean, standard deviation, minimum, and maximum (in tables), and as median with interquartile range in boxplots. Categorical data are reported as proportions. In boxplots, the median is shown by a black line, IQR by colored boxes, and whiskers indicate the range.

For continuous variables, based on the Shapiro test results from rstatix package, normally distributed data were analysed using linear mixed models (LMMs) fitted via the lmer function (lme4 package v1.1.35.5), while non‐normal data were modelled with generalized linear mixed models (GLMMs) using a Gamma distribution and log‐link through glmer. In both analyses, the fixed effects formula used was “time + BMI + sex + time|BMI + time|sex”, where BMI was considered as groups divided as it: Lean (BMI < 25), Overweight (25 ≤ BMI < 30), and Obese (BMI ≥ 30). The random effect in both models was the individual to account for repeated measures and to control individual variability. Also, post hoc comparisons were conducted using estimated marginal means (emmeans package v1.10.5), reporting estimated means and CI for each comparison, with *p*‐values adjusted (P) via the Benjamini–Hochberg method. Spearman's rank correlations were calculated using rcorr (Hmisc), with BH‐adjusted *p*‐values and hierarchical clustering (hclust) to order the correlation matrix. Heatmaps display only significant (adjusted *p*‐value < 0.05) coefficients.

No a priori power calculation was performed; the sample size reflects feasibility for a single‑arm pilot intended to estimate effect sizes and variance components to inform a subsequent randomized controlled trial.

## 3 Results

### 3.1 Participant Disposition, Adherence, and Safety

**FIGURE 1:**
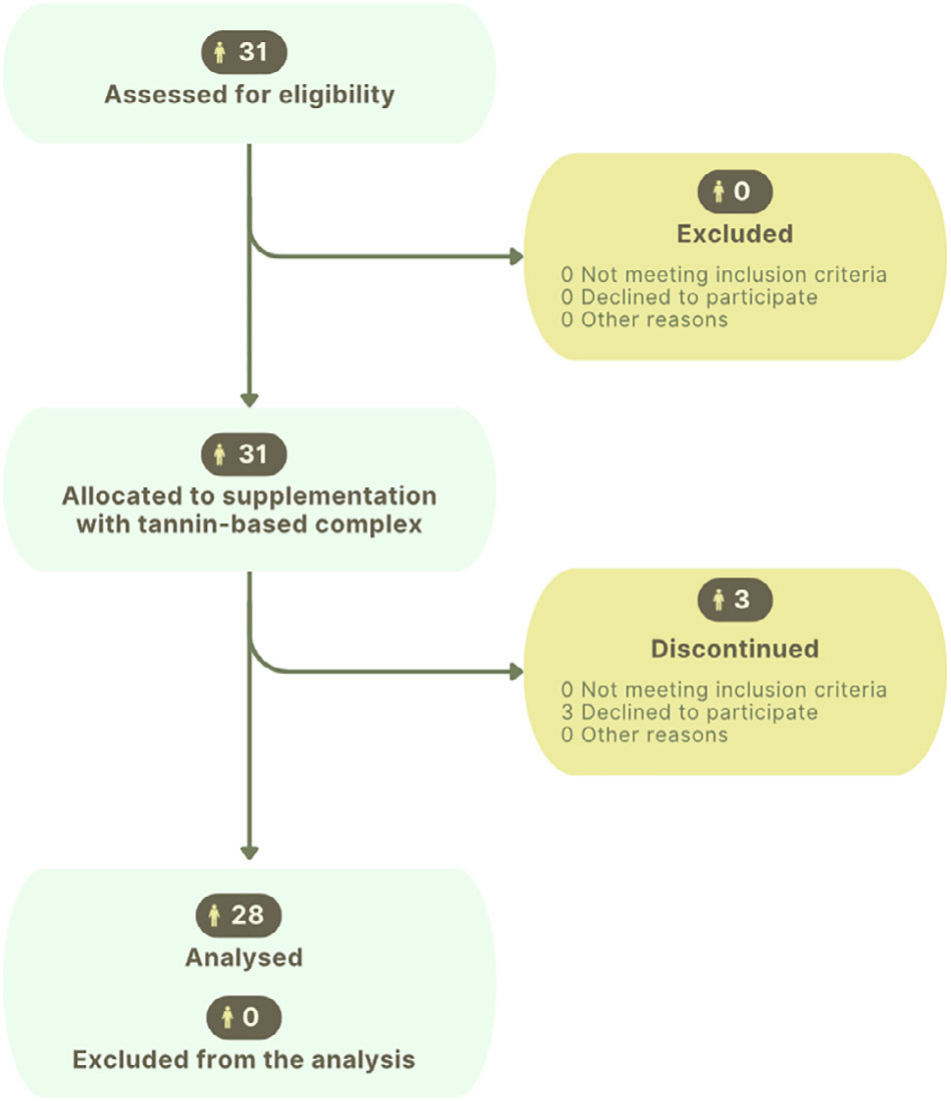
Flowchart, according to the CONSORT (Consolidated Standards of Reporting Trials) guidelines, illustrating the stages of enrolment, allocation, follow‐up and analysis of participants in a clinical trial.

The study followed the flowchart in Figure 1, in line with CONSORT PRO guidelines [24]. Of the original 31 subjects, three discontinued. Participants with IBS‐D had a median age of 43 years (range 26–76), and 71% were female. Based on BMI, participants included 13 normal‐weight, 7 overweight, and 8 obese patients, with a mean BMI of 27.82 ± 6.71. Baseline data are shown in Table 1. The mixed‐effect model analysed time, sex, BMI, and their interactions.

**TABLE 1:** Baseline (T0) demographic data of the study population, including the median, range, mean, and standard deviation for the subjects by sex. Total number of participants are shown also disaggregated by sex and BMI category.

	Females (*n* = 20)	Males (*n* = 8)	Total (*n* = 28)
Age (years)	49 [26–76] 51.15 ± 17.19	36.5 [30–75] 43 ± 17.14	43 [26–76] 48.82 ± 17.26
BMI	24.55 [19.8–42.2] 27.84 ± 7.44	26.45 [22.3–37.2] 27.77 ± 4.84	25.6 [19.8–42.2] 27.82 ± 6.71
*Normal weight*	*n* = 11	*n* = 2	*n* = 13
*Overweight*	*n* = 3	*n* = 4	*n* = 7
*Obese*	*n* = 6	*n* = 2	*n* = 8

Adherence to the dosing regimen was monitored by daily telephone calls and was satisfactory in all completers. No adverse events were reported during the study.

### 3.2 Primary Outcomes

**FIGURE 2:**
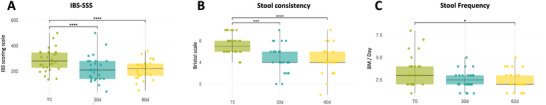
(A) Clinical symptoms (IBS Severity Score), (B) Stool consistency, and (C) Stool frequency shifts along time. Significant improvement in IBS severity (IBSSS) scores and Stool consistency (Bristol scale) after tannin supplementation, with scores decreasing at 30d and 60d. Stool frequency also improved significantly after tannin supplementation at 60d.

The IBS Severity Scoring System (IBS‑SSS) showed a significant reduction following 60 days of supplementation, with an estimated mean reduction of 70 points (95% CI: 34–107; *p* < 0.0001), exceeding the commonly accepted threshold for clinically meaningful improvement of 50 points (Figure 2A). The direction and magnitude of change indicate a shift toward lower symptom burden across the cohort, with observed mean IBS‐SSS scores decreasing from severe‐moderate (288 ± 90.3) to moderate‐mild (214 ± 73.7) at 60d (Table 2). The interaction time × BMI resulted significant (*p* = 0.0195) with a greater symptoms reduction observed for obese individuals (−36.36%), who among all patients were found to exhibit higher initial IBS‐SSS values. No significant effects were found for sex or time × sex (Table S1).

**TABLE 2:** Descriptive statistics, including the mean, standard deviation, and range, for the subjects over time (T0, 30d, and 60d) across the three variables selected as primary outcomes. Additionally, it reports the P‐values derived from model analysis for the various tested effects and their interactions.

Variable	T0	30d	60d	*p*‐value				
				Time	BMI	Sex	Time × BMI	Time × Sex
**IBS‐SSS**	288 ± 90.3 (135 ‐ 500)	218 ± 102 (40 ‐ 500)	214 ± 73.7 (50 ‐ 360)	0.001	0.812	0.076	0.02	0.778
**Stool consistency** **(Bristol scale)**	5.64 ± 0.911 (4 ‐ 7)	4.38 ± 1.27 (2 ‐ 7)	4.19 ± 1.27 (1 ‐ 7)	<0.0001	0.162	0.989	0.251	0.128
**Stool frequency (BM/Day)**	3.39 ± 1.71 (1 ‐ 8)	2.54 ± 1.03 (1 ‐ 5)	2.41 ± 1.08 (1 ‐ 5)	0.018	0.029	0.383	0.981	0.510

Stool consistency improved significantly by 30d (with estimated mean change of 1.22; 95% CI: 0.56 to 1.89; *p* < 0.0001, Figure 2B) and this effect was maintained by 60d (with estimated mean change of 1.38; 95% CI: 0.72 to 2.05; *p* < 0.0001), with no notable differences due to sex or BMI. Mixed‐effects model analysis indicated a significant reduction in daily bowel movements from baseline to 60d (estimated mean change ‐0.84 movements/day; 95% CI: ‐0.08 to ‐1.61; *p* = 0.027), with observed values dropping from 3.39 ± 1.71 (T0) to 2.41 ± 1.09 (60d) (Figure 2C), with obese subjects having higher frequencies (*p* = 0.023). Sex and interaction effects did not reach significance. Table 2 presents descriptive statistics and model results by time, sex, BMI, and their interactions.

### 3.3 Secondary Outcomes

The secondary outcomes evaluated the impact on intestinal symptoms and quality of life in IBS‐D patients, along with related mechanisms such as inflammation, SCFA production, and intestinal permeability.

#### 3.3.1 Intestinal Symptoms

**FIGURE 3:**
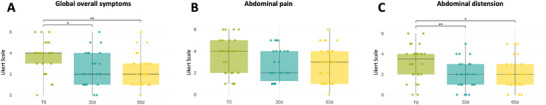
Shifts along time of intestinal symptoms evaluated as (A) global overall symptoms (B) abdominal pain and (C) abdominal distension, through a 7‐points Likert scale. Significance brackets display post‐hoc test results: “*” denotes *p* < 0.05 and “**” denotes *p* < 0.01.

Global overall symptom severity, abdominal distension, and pain were tracked during the study. Table 3 shows means and ranges for T0, 30d, and 60d. Treatment significantly reduced overall symptom severity and abdominal distension (*p* = 0.002, *p* = 0.0042, Figures 3A, C), with notable improvement by 30d. Tannin supplementation lowered symptom intensity by over one point on the 7‐point Likert scale. Abdominal pain decreased, though not statistically significant (Figure 3B). Time × sex analysis revealed greater improvements in pain (*p* = 0.036) and distension (*p* = 0.040) in women (Table S2).

**TABLE 3:** Descriptive statistics, including the mean, standard deviation, and range, for some of the secondary outcome variables, for the subjects over time (0d, 30d, (when applicable) and 60d). Additionally, it reports the *P*‐values derived from model analysis for the various tested effects and their interactions.

Variable	0d	30d	60d	*p*‐value				
				Time	BMI	Sex	Time × BMI	Time × Sex
Global overall symptoms (Likert scale)	3.64 ±1.5 (0 ‐ 6)	2.54 ± 1.61 (0 ‐ 6)	2.41 ± 1.53 (0 ‐ 6)	0.002	0.859	0.73	0.093	0.252
Abdominal pain (Likert scale)	3.5 ± 1.67 (1 ‐ 6)	2.65 ± 1.47 (1 ‐ 5)	2.7 ± 1.71 (0 ‐ 6)	0.315	0.778	0.522	0.683	0.036
Abdominal distension (Likert scale)	3.14 ± 1.65 (0 ‐ 6)	1.92 ± 1.38 (0 ‐ 5)	1.96 ± 1.56 (0 ‐ 5)	0.004	0.299	0.275	0.903	0.040
PHQ‐15	14.7 ± 5.38 (6 ‐ 30)	11.1 ± 5.41 (1 ‐ 27)	10.3 ± 4.26 (2 ‐ 20)	<0.001	0.866	0.02	0.597	0.005
Anxiety (HADS)	10.9 ± 4.55 (4 ‐ 20)	9.46 ± 4.54 (2 ‐ 18)	9.41 ± 4.44 (3 ‐ 18)	0.429	0.029	0.658	0.479	0.053
Depression (HADS)	7.32 ± 3.94 (0 ‐ 14)	6.54 ± 4.11 (1 ‐ 15)	6.19 ± 4.31 (0 ‐ 15)	0.368	0.546	0.946	0.443	0.339
Stress (PSS)	29 ± 9.26 (8 ‐ 41)	26.6 ±9.7 (8 ‐ 43)	26.4 ± 9.89 (9 ‐ 43)	0.003	0.253	0.713	0.672	0.213
IL‐1b (pg/mL)	0.62 ± 0.21 (0.34 ‐ 1.32)		0.66 ± 0.29 (0.13 ‐ 1.51)	0.533	0.441	0.273	0.528	0.047
IL‐1ra (pg/mL)	226 ±139 (0 ‐ 574)		245 ±133 (0 ‐ 547)	0.682	0.004	0.443	0.089	0.901
IL‐9 (pg/mL)	8.6 ± 0.83 (7.23 ‐ 10.2)		7.87 ±1.74 (0 ‐ 9.59)	0.068	0.785	0.973	0.649	0.944
MIP‐1a (pg/mL)	0.843 ± 0.64 (0 ‐ 2.08)		1.6 ± 2.01 (0 ‐ 8.84)	0.080	0.006	0.092	0.014	0.069
Zonulin (ng/mL)	36.05 ± 5.16 (26.84 ‐ 50.06)		24.87 ± 3.92 (16.17 ‐ 32.65)	<0.001	0.080	0.526	0.612	0.512
Butyrate (mM)	1.54 ± 1.06 (0 ‐ 4)		2.96 ± 1.59 (1 ‐ 7)	0.001	0.762	0.141	0.967	0.477
Propionate (mM)	5.82 ± 5.38 (1 ‐ 24)		2.95 ± 1.33 (1 ‐ 6)	0.017	0.275	0.497	0.135	0.520
Acetate (mM)	13.7 ±6.7 (3 ‐ 28)		15.3 ± 4.19 (7 ‐ 23)	0.547	0.910	0.728	0.585	0.214
Formate (mM)	0.543 ± 0.605 (0 ‐ 3)		0.179 ± 0.23 (0 ‐ 1)	0.239	0.851	0.292	0.001	<0.001

#### 3.3.2 Psychological Symptoms

**FIGURE 4:**
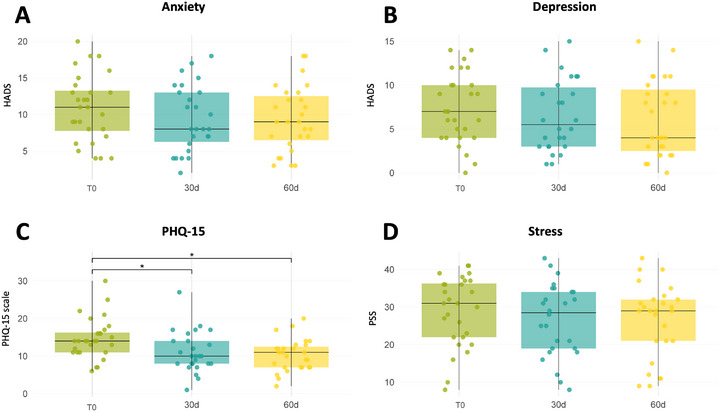
Psychological symptoms evaluated through questionaries. (A) Anxiety and (B) Depression levels measured with HADS showed a non‐significant decrease (*P*=0.429 and *P*=0.368, respectively). (C) PHQ‐15 scores demonstrated a significant reduction at both 30d and 60d (*P* <0.0001). D) Stress levels assessed using PSS showed a significant shift over time (*P*=0.0026). Significance brackets indicate post‐hoc test results: “*” denotes *p* < 0.05, “**” denotes *p* < 0.01, “***” denotes *p* < 0.001, and “****” denotes p < 0.0001.

The gut‐brain axis's effect on psychological measures after tannin treatment was evaluated with several questionnaires (Table 3). PHQ‐15 scores decreased significantly over time, indicating a reduction in somatic symptom severity (*p* < 0.0001, Figure 4C). Sex served as a modifying factor (*p* = 0.0292), with women exhibiting greater improvement in PHQ‐15 scores compared to men, indicating that the intervention was more effective in reducing somatic symptoms among females. Additionally, the time × sex interaction resulted statistically significant (*p* = 0.0046Tables S1, S2).

Clinically, these results indicated a reduction in symptom severity from borderline severe to lower moderate (Table 3). In particular, women's scores decreased from 16.45 ± 5.01 (severe) to 11.11 ± 6.01 at 30 days and 10 ± 4.26 at 60 days, approaching mild levels (Tables S1, S2).

Depression and anxiety (HADS) trended better but were not statistically significant, though BMI did influence anxiety levels (p = 0.0295), with overweight individuals showing more anxiety than obese ones (Table S2). Stress (PSS) decreased significantly (*p* = 0.0026, Figure 4D) with scores moving from high to moderate levels (Table 3) representing both a statistically and clinically significant reduction.

#### 3.3.3 Inflammatory state

27 different human cytokines, chemokines and growth factors were analyzed. No significant changes were identified in the panel of biomarkers of inflammation tested.

Only in a few cases a trend was found, as in the case of IL‐9, which showed a decrease from 8.6 ± 0.83 pg/ml to 7.87 ± 1.74 pg/ml (*P* = 0.0677). Significant differences were observed for IL‐1ra and MIP‐1a due to the influence of sex and/or BMI (Table 3). Further detailed results can be found in Tables S1 and S2.

#### 3.3.4 Gut Microbiota Functionality

**FIGURE 5:**
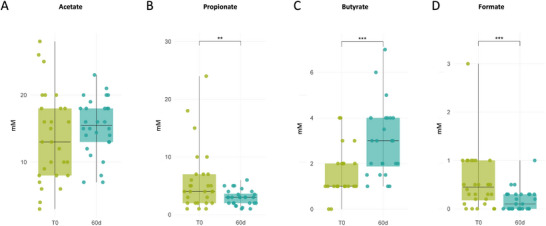
Short Chain Fatty Acids (SCFAs) levels at baseline and 60d. (A) Acetate, (B) propionate, (C) butyrate and D) formate levels are showed in mM, being propionate and formate significantly decreased (*p* = 0.017 and *p* = 0.2392) at 60d after tannin supplementation, while butyrate increased significantly at 60d (*p* = 0.0004). Formate decreased non significantly at 60d (*p* = 0.24), although post hoc test indicated a significant effect of treatment. Significance brackets display post‐hoc test results: “**” denotes *p* < 0.01 and “***” denotes *p* < 0.001.

Gut microbiota metabolism was monitored by measuring the three principal SCFA concentration: acetic, propionic, and butyric acid (Figures 5A–C). In IBS‐D patients, tannin treatment SCFA levels while maintaining the typical ratio: acetate > propionate > butyrate. Acetate and butyrate increased (*p* = 0.0004), propionate decreased significantly (*p* = 0.0170) from T0 to 60d (Table 3).

Formic acid levels also declined after treatment; although not significant by LMM (*p* = 0.2392), a post‐hoc test showed a significant time effect (*p* < 0.0001). Significant combined effects of time × BMI and time × sex were observed (*p* = 0.0004, *p* < 0.0001), indicating greater reductions in normal weight (−63%), obese individuals (−20%), and males (−18%) (Tables S1 and S2).

#### 3.3.5 Intestinal Permeability

**FIGURE 6:**
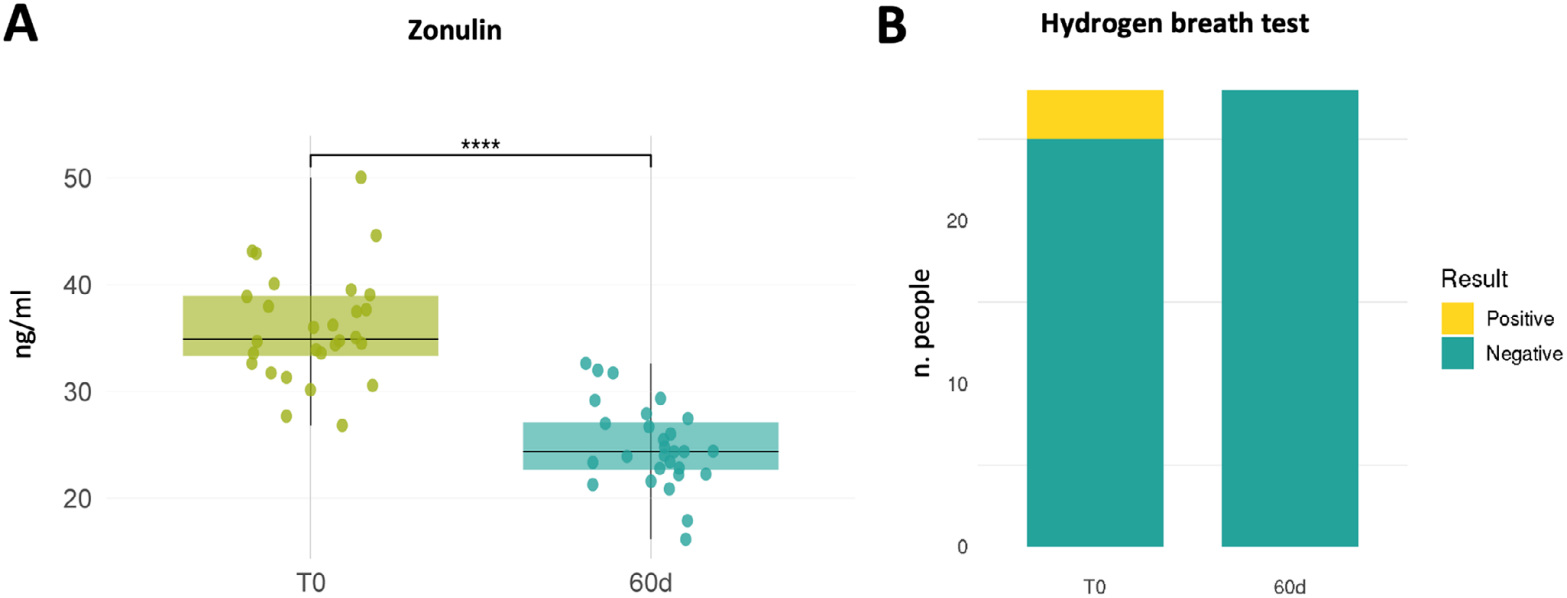
(A) Zonulin levels showed a significant reduction at 60d compared to baseline (*p* < 0.0001). (B) Hydrogen breath test results showed complete negativisation at 60d, with three subjects testing positive at baseline.

Serum zonulin levels, indicating intestinal permeability, significantly decreased from 36.05 ± 5.16 ng/mL to 24.87 ± 3.92 ng/mL over time (*P* < 0.0001) (Figure 6A). At 60d, all patients' levels were within the normal range.

#### 3.3.6 Hydrogen Breath Test Results Showed That

Out of 28, 3 IBS‐D patients (10.71%) resulted H‐positive at the beginning of the study (Figure 6B). After the 60‐day supplementation with the tannin complex, all patients tested negative.

### 3.4 Correlation Analysis

**FIGURE 7:**
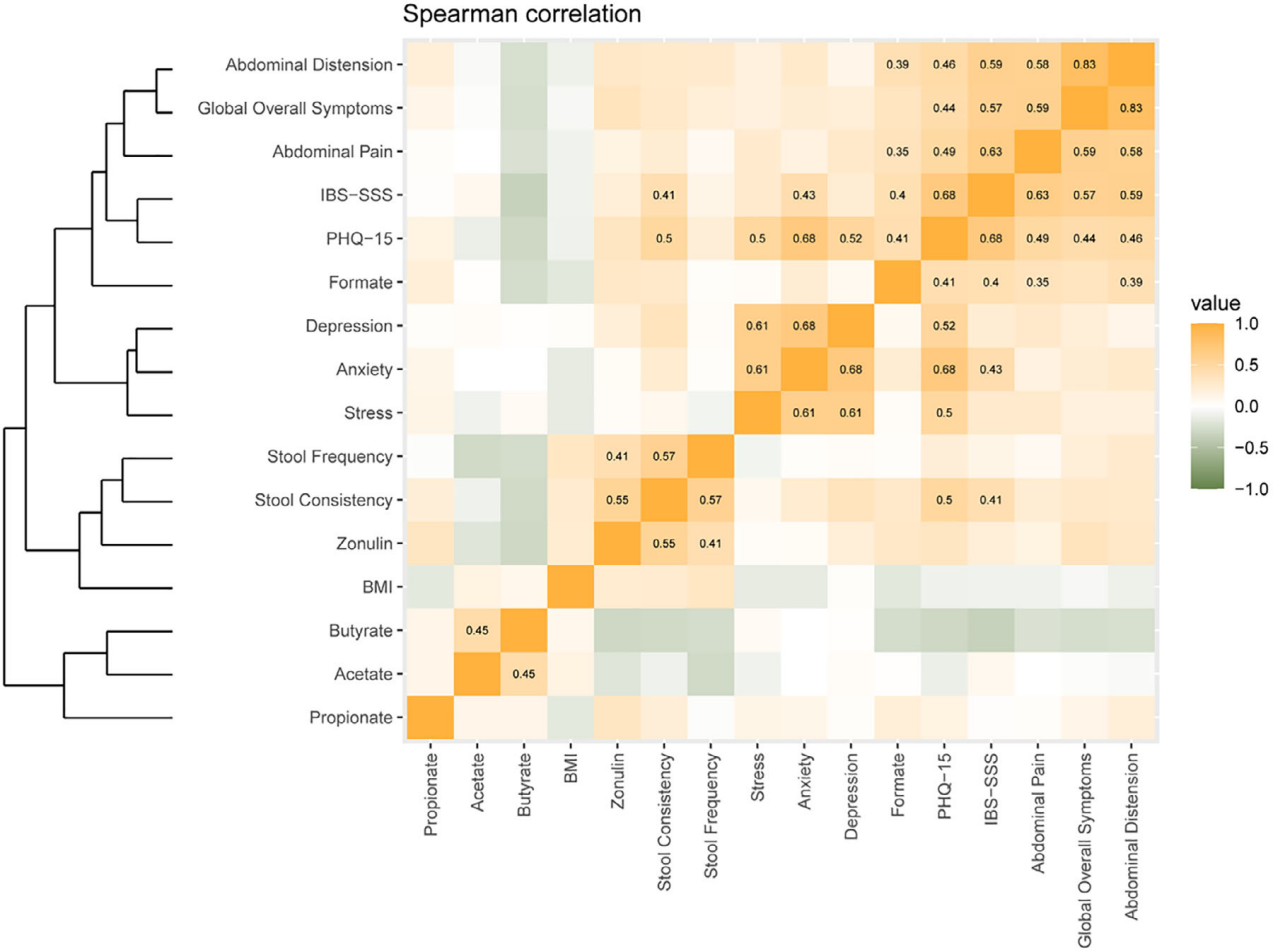
Clustered image map of Spearman correlation analysis of selected variables. Colour represents the r value between variables. Only significant (*p*‐value < 0.05) correlations display the *r*‐value.

From the study emerged a large number of correlations, but only a few presented a stronger effect. The dendrogram in Figure 7 shows how the variables examined can be grouped from top to bottom: 1) intestinal‐related symptoms together with indicators of general IBS symptomatology (IBS‐SSS and PHQ‐15) and formate, 2) mental health‐related symptoms (depression, anxiety and stress), 3) evacuation‐related variables (stool consistency, frequency of deposition and intestinal permeability) together with BMI, 4) three main SCFAs (acetate, propionate and butyrate). The groupings with the dendrogram also herald a positive correlation between the above‐mentioned clusters of variables.

The highest number of correlations were recorded for PHQ‐15 and IBS‐SSS, which showed a positive relationship with almost all indicators of mental and intestinal symptoms.

Formate was notably associated with IBS symptom measures, abdominal distension, and pain. Zonulin, Bristol score, and stool frequency were positively related. Intestinal permeability (serum zonulin) correlated significantly with stool consistency and frequency (*r* = 0.55 and *r* = 0.41). Stool consistency also affected general symptom scores such as PHQ‐15 (*r* = 0.5) and IBS‐SSS (*r* = 0.41).

While the main SCFAs did not significantly correlate with IBS‐D symptomatology, acetate and butyrate tended to correlate negatively with symptoms and permeability, whereas propionate showed positive correlations.

## 4 Discussion

This exploratory study in 28 adults with IBS‐D evaluated the clinical impact and mechanistic signatures of an eight‐week, twice‐daily supplementation with a tannin‐based phytocomplex from quebracho and chestnut. The intervention was associated with a consistent reduction in global IBS‐SSS and with improvements in bowel habits, including fewer diarrhoeal evacuations and more formed stools. Secondary domains (global overall symptoms, abdominal pain, distension) showed also a coherent change, indicating a pattern on the intestinal phenotype that is consistent with the gut‐brain axis framework.

At the intestinal interface, tannins can strengthen barrier function by modulating tight junction scaffolding (e.g., occludin, claudin‐1), thereby limiting paracellular permeability [25]. They are also reported to temper NF‐κB activation and downstream pro‐inflammatory transcription, providing a biologically plausible link to lower intestinal discomfort and improved motility patterns [26]. The clinical signal observed here, coupled with the decline in serum zonulin is consistent with these mechanisms and is compatible with the barrier‐centric component of IBS‐D pathophysiology.

Because IBS is shaped by bidirectional signalling between the gut and the brain, psychological outcomes are not ancillary but integral to disease expression [1, 2]. In our cohort, stress (PSS) and somatisation (PHQ‐15) were lower alongside gastrointestinal endpoints. Mechanistically, short chain fatty acids (SCFAs), notably butyrate and acetate, can cross the gut barrier, engage neuroimmune pathways, and influence central and enteric circuits implicated in stress responsivity and affective tone [27]. The parallel changes in psychological and intestinal domains therefore supports an integrative gut‐brain interpretation rather than isolated symptomatic relief.

This comprehensive perspective is consistent with prior work emphasizing that symptom burden and quality of life in IBS hinge on both gastrointestinal and psychological dimensions [28]. Psychosocial stressors may trigger or amplify bowel symptoms, reinforcing a vicious cycle between mind and gut; such dynamics have tangible consequences for daily functioning and healthcare utilization [29]. Stress‐related surges in cortisol and pro‐inflammatory cytokines can exacerbate barrier dysfunction, whereas tannin‐mediated attenuation of inflammatory signalling has been reported and offers a plausible counterweight at the mucosal level [30].

Contemporary care pathways increasingly promote holistic strategies that combine dietary, behavioral, and targeted symptomatic approaches rather than focusing on single nodes of the disorder [31]. Within this paradigm, a tannin‐based phytocomplex is nutritionally relevant because it interfaces with several drivers of IBS‐D, including microbial ecology, barrier integrity, motility, and stress biology, within one intervention. Diet remains foundational, and emerging analyses underline that dietary modulation can differentially affect IBS subtypes [32], yet the present data suggest that tannin supplementation may add value where permeability, SCFA profiles, and stress‐related axes are active contributors.

Our previous investigations with the same formulation reported favorable modulation of gut microbiota composition and activity in IBS‐D, with potential downstream effects on gut‐brain symptomatology [33]. This is salient because IBS‐D often features altered SCFA profiles due to depletion of producer taxa and disruption of key biosynthetic pathways, with consequences for epithelial homeostasis and motility [34, 35, 36]. After 60 days, we observed an increase in acetate and butyrate and a reduction in propionate, shifts that are often described as aligning with a health‐associated SCFA configuration in literature and that correlate with concurrent trends in symptoms. Recent reviews reiterate the therapeutic relevance of SCFA rebalancing in gut health and its pharmacological implications [37]. Within this landscape, tannins and other polyphenols can influence microbial enzymatic routes (e.g., acetyl‐CoA and propionate kinase–dependent pathways), offering a mechanistic rationale for the SCFA profile observed here [37]. Furthermore, the reduction in formate, which correlated with improvements in intestinal symptoms, IBS‐SSS, and somatization, resonates with literature linking one‐carbon fermentation products to methanogenesis and inflammatory tone in the gut milieu [37].

Small intestinal bacterial overgrowth (SIBO) represents an additional, frequently overlapping driver of IBS‐D. Despite validation, glucose hydrogen breath test shows moderate sensitivity (often ∼60%) and variable specificity, so we interpreted results cautiously in clinical context [38]. In our cohort, 10% of participants were hydrogen‐positive at baseline; all were negative at eight weeks. This normalization was observed, considering the documented prevalence of hydrogen‐producing SIBO in IBS‐D and its association with diarrhea, bloating, and abdominal pain [39, 40, 41]. Tannins possess antimicrobial properties, including the capacity to bind microbial proteins and enzymes and to interfere with growth and adhesion, a profile consistent with the observed breath test changes [42]. Although breath testing has limitations and does not capture the full complexity of small bowel ecology, the concordance between SIBO resolution and symptomatic changes supports compatibility with microbial load and fermentation patterns as potential contributors to the overall response.

Barrier restoration emerged as a recurrent finding. The decline in serum zonulin, together with symptomatic changes is compatible with better containment of luminal factors and reduced epithelial leakiness, both of which are increasingly recognized in disorders of gut‐brain interaction [43, 44]. While part of the diarrheal change might reflect the astringent properties attributed to tannins and related barrier forming agents [45, 46, 47], effects on motility likely extend beyond a purely topical mechanism. Ex vivo work indicates that the individual ingredients in this phytocomplex can modulate intestinal contractility through distinct actions on smooth muscle, offering a plausible pathway that could explain stool form and frequency changes [48].

Shifts in circulating cytokines did not reach statistical significance after adjustment for multiple testing. However, this does not exclude a potential anti‑inflammatory effect, as normal baseline levels, low systemic inflammation, or a short intervention period may have limited detectable changes. Larger, controlled trials are needed to confirm any effects, especially given variable cytokine patterns and results reported in the literature across different contexts (e.g., IBS subtypes, sex, comorbidities, therapies) [49].

Exploratory pattern suggested, women and participants with higher BMI may have shown greater responsiveness on selected variables, for potential biological reasons. Differences between sexes in the gut–brain axis, influenced partly by the effects of oestrogen on epithelial and immune responses as well as by sex‐specific microbiome patterns, may affect how individuals respond to a tannin‐based intervention that targets barrier function or the microbiota [50, 51]. In parallel, obesity is often accompanied by dysbiosis, altered bile‑acid and short‑chain fatty‑acid signalling, and increased intestinal permeability, which may allow more potential for a for barrier‑ and microbiota‑targeted supplementation [52, 53]. However, in our preliminary analyses neither sex or BMI emerged as major modifiers of response, such hypotheses are therefore speculative at this stage and based only on initial feasibility results. Comprehensive, stratified studies are required to validate whether responses to this tannin‐based intervention vary by sex and BMI, as well as to identify microbiome or host markers that reliably predict outcomes.

Correlation analysis provided additional insight into the interplay between intestinal and psychological domains. Significant associations were observed between serum zonulin and bowel habit metrics, supporting the link between barrier integrity and motility. Formate levels correlated with IBS‐SSS, and somatization scores assessed by PHQ‐15, suggesting that microbial metabolites may influence both gastrointestinal and extra‐intestinal symptoms. Hierarchical clustering grouped PHQ‐15 somatization and IBS‐SSS with gastrointestinal symptom domains, reinforcing the concept of a bidirectional gut–brain axis.

Complementing the clinical and biomarker signals described above, we outline a potential intestine‑to‑brain link within the gut–brain axis: by tightening epithelial junctions and lowering zonulin, tannins may limit microbial translocation, and thereby reduce immune signals reaching the brain, without necessarily implying a demonstrated anti‑inflammatory effect in our dataset. In parallel, tannin‑related shifts toward SCFA‑producing taxa and higher SCFAs are consistent with enteroendocrine and vagal pathways that relay luminal cues to the central nervous system; notably, butyrate can modulate tight‑junction programs and mucosal immune tone in a context‑dependent manner in humans, offering a plausible, non‑causal rationale for the observed psychological readouts. Considering that polyphenol‑rich diets have been associated with lower circulating “zonulin‑like” signals in randomized trials and by triangulating interpretation with convergent endpoints (stool form/frequency, SCFAs) our findings delineate credible intestine‐to‐brain routes that remain hypothesis‑generating and require confirmation in randomized, controlled studies [54, 55, 56].

This study has limitations inherent to its exploratory design. The single‐arm, open‐label format without randomization precludes causal inference and may allow placebo‐related effects. The absence of a control group represents also a limitation that prevents attributing the observed changes specifically to the intervention. However, mechanistic consistency across clinical, psychological, and biomarker domains mitigates this concern. The modest sample size and 60‐day timeframe limit generalizability and long‐term insights yet were sufficient to detect coherent signals in IBS‐SSS, PHQ‐15, zonulin, SCFA profiles, and breath testing. Diet was not strictly controlled, introducing potential confounding, but adherence monitoring and biomarker trends suggest a genuine intervention effect. Despite these constraints, strengths include high compliance, absence of adverse events, and an integrated multi‐domain assessment supporting the plausibility of a gut‐brain axis targeted nutritional approach.

To further strengthen the validity of these observations, a double‐blind, placebo‐controlled trial with a larger, prospectively calculated sample size, and potentially incorporating dietary control (i.e., a FODMAP approach), may help to re‑examine the clinical co‑primary endpoints and the mechanistic panel, and include pre‑specified sex/BMI stratification to test effect heterogeneity suggested here.

## 5 Conclusion

Supplementation with a tannin‐rich phytocomplex derived from quebracho and chestnut was linked to improvements across gastrointestinal and psychological domains in IBS‐D, alongside signals compatible with gut–brain axis engagement (microbial metabolites, epithelial‑barrier indices, and stress‑related measures). These data remain preliminary owing to the single‑arm, feasibility design, but they offer effect‑size estimates and mechanistic leads to inform the design of definitive trials, guide dose and duration optimization, and explore integration with personalized diet and lifestyle.

## Funding

This study was sponsored by University Hospital of Universidad de Buenos Aires “Hospital de Clínicas José de San Martín” (Argentina), which was responsible for protocol approval, patient recruitment, and data analysis. Silvateam S.p.A. provided the tannin‐based supplement used in the trial but had no role in data interpretation. No additional external funding was received.

## Conflicts of Interest

Silvia Molino is employed by Silvateam S.p.A. (R&D Unit), which provided the tannin‐based supplement for this study and is listed as a collaborator on ClinicalTrials.gov. Alberto Lerma was also employed by Silvateam S.p.A. during part of the study period. The sponsor (Hospital de Clínicas José de San Martín) retained full responsibility for study design, data collection, and analysis. All other authors declare no conflicts of interest.

## Informed Consent Statement

Informed consent was obtained from all subjects involved in this study.

## Supporting information




**Supporting File**: mnfr70451‐sup‐0001‐SupMat.xlsx.

## Data Availability

The data that support the findings of this study are available from the corresponding author upon reasonable request. The data will be provided after its de‐identification, in compliance with applicable privacy laws, data protection, and requirements for consent and anonymization.
